# Diagnostic Potential of Two Novel Biomarkers for Neuromyelitis Optica Spectrum Disorder and Multiple Sclerosis

**DOI:** 10.3390/diagnostics13091572

**Published:** 2023-04-27

**Authors:** Ting Xu, Yijun Shi, Guanghui Zheng, Guojun Zhang

**Affiliations:** 1 Laboratory of Beijing Tiantan Hospital, Capital Medical University, Beijing 100070, China; 18813186407@163.com (T.X.);; 2 NMPA Key Laboratory for Quality Control of In Vitro Diagnostics, Beijing 100070, China; 3 Beijing Engineering Research Center of Immunological Reagents Clinical Research, Beijing 100070, China

**Keywords:** MS, NMOSD, IGFBP7, LAMP2, diagnosis, prediction

## Abstract

Background: Currently, no tests can definitively diagnose and distinguish neuromyelitis optica spectrum disorder (NMOSD) from multiple sclerosis (MS). Methods: Initially, cerebrospinal fluid (CSF) proteomics were employed to uncover the novel biomarkers that differentiate NMOSD from MS into cohorts of 10 MS and 10 NMOSD patients. Subsequently, screening biomarkers were validated using an enzyme-linked immunosorbent assay method and CSF and serum samples from 20 MS patients, 20 NMOSD patients, 20 non-inflammatory neurological controls, and 20 healthy controls. Results: In study cohort, insulin-like growth factor-binding protein 7 (IGFBP7) and lysosome-associated membrane glycoprotein 2 (LAMP2) were screened. In validation cohort, serum and CSF IGFBP7 not only exhibited higher levels in MS and NMOSD patients than controls, but also had greatest area under the curve (AUC, above or equal to 0.8) in MS and NMOSD diagnoses. Serum IGFBP7 (0.945) and CSF IGFBP7 (0.890) also had the greatest AUCs for predicting MS progression, while serum LAMP2 had a moderate curve (0.720). Conclusions: IGFBP7 was superior in diagnosing MS and NMOSD, and IGFBP7 and serum LAMP2 performed exceptionally well in predicting the MS progression. These results offered reasons for further investigations into the functions of IGFBP7 and LAMP2 in MS and NMOSD.

## 1. Introduction

Multiple sclerosis (MS) is the most common chronic inflammatory, demyelinating, and neurodegenerative disease of the central nervous system, affecting young adults [1,2]. Significant risk factors in its pathogenesis include inflammation and genetic or environmental variables [3]. The four main types of multiple sclerosis are clinically isolated syndrome (CIS), relapsing-remitting MS (RRMS), secondary progressive MS (SPMS), and primary progressive MS (PPMS). Depending on the extent and location of nerve fiber damage in the central nervous system, patients with MS may possess a wide range of signs and symptoms. Some individuals with severe MS may be incapable of walking independently or ambulating at all. There are also people who experience extended periods of remission without suffering any new symptoms.

Neuromyelitis optica spectrum disorder (NMOSD), formerly considered to be a subtype of multiple sclerosis (MS), is now recognized as a separate disorder that can be confused with MS [4]. In clinical practice, distinguishing between these two disorders is of the utmost importance. This is because NMOSD requires treatment long-term immunosuppressive medicine to prevent a devastating recurrence, whereas MS therapies such as interferon-β and nazumab may worsen NMOSD [5,6]. Currently, NMOSD and MS are mostly diagnosed using clinical parameters and imaging examinations. The new NMO criteria from 2015 and the revised McDonald criteria from 2017, the guidelines that are used to diagnose NMOSD and MS, highlight the requirements for MR imaging [7,8]. Researchers are actively researching the use of body fluid biomarkers for the diagnosis of these two disorders [9,10]. A subset of people with NMOSD display anti-myelin oligodendrocyte glycoprotein (MOG) antibodies in their blood. Seventy-five percent of patients display anti-aquaporin 4 (anti-AQP4) antibodies. Patients who test positive for anti-AQP4 NMOSD are more likely to display oligoclonal IgG protein in 20% of cases [3]. It seems that all examinations and clinical, blood, and CSF tests do not appear to permit the indefinite separation of NMOSD from MS.

With the backing of mass spectrometry and bioinformatics technologies, a high-resolution and high-accuracy proteomics technique has been established to identify new MS and NMOSD biomarkers [11,12] in CSF [13,14,15,16,17], sera [18], and urine [19]. These investigations demonstrate the considerable diagnostic potential of this approach in clinical diagnostics. Hence, we initially employed TMT labeling in conjunction with high-resolution LC-MS/MS analysis to identify the differential proteome p4esent in the CSF of MS and NMOSD patients for this investigation. The proposed biomarkers were further verified using ELISA in a separate cohort. This work helped us to uncover novel biomarkers of MS and NMOSD and provided a basis for further investigation into the functions of these proteins in the pathogenesis of MS and NMOSD.

## 2. Materials and Methods

### 2.1. Patients

From 2018 to 2021, a number of individuals registered at the Beijing Tiantan Hospital of Capital Medical University and 100 were enrolled in this study. The research consisted of two phases. Initially, potential biomarkers were found using discovery samples of 10 MS and 10 NMOSD patients. The identified biomarkers were subsequently validated using a validation cohort consisting of 20 cases of MS patients (7 relapsing remitting multiple sclerosis (RRMS) and 13 secondary progressive multiple sclerosis (SPMS)), 20 cases of NMOSD patients, 20 cases of non-inflammatory neurological controls (NINC), and 20 cases of healthy controls (HC).

The inclusion criteria for patients with NMOSD were as follows: (1) age ≥ 18 years; and (2) diagnosis according to the 2015 diagnostic criteria for NMOSD [7]. Inclusion criteria for MS patients were: (1) age ≥ 18 years; and (2) diagnosis according to the 2017 updated McDonald criteria [8]. Exclusion criteria were as follows: (1) age < 18 years; (2) major disorders of the heart, liver, kidney, and other critical organs or blood vessels; and (3) complications owing to a malignant tumor. 

Our study was performed per the Declaration of Helsinki and authorized by the ethical committee of the Tiantan Hospital of Capital Medical University (Ethics Committee document number: KYSQ 2020-092-01). All patients provided a signed informed consent.

### 2.2. Quantitative CSF Proteomics Analysis Using TMT Technology

All individuals underwent a lumbar puncture under local anesthesia with lidocaine, and 3 mL of CSF was collected and stored at −80 °C in several 1.5 mL Eppendorf tubes before use. As recommended by the ProteoMiner protein enrichment kit, the protein concentration was determined with the BCA assay following the removal of abundant protein (Bio-Rad, Hercules, CA, USA).

The human CSF samples (100 μL/sample) were digested using the filter-aided sample preparation (FASP) method with minimal modifications [20]. Each sample was combined with 50 μL of lysis solution (4% SDS, 0.1 M DTT in 0.1 M Tris-HCl, pH 7.6), passed to a 10 K filter (Pall Corporation), and centrifuged for 10 min at 10,000× *g* and 20 °C. After adding 200 μL of urea (UA) buffer (8M urea, 0.1 M Tris-HCl, pH8.5), the sample was centrifuged at 14,000× *g* for further 20 min. The concentrate was then combined with 200 μL of 100 mM IAA in a UA buffer and incubated for a further 40 min at room temperature in the dark, after which the IAA was removed by centrifugation at 14,000× *g* for 20 min. After dilution with 200 μL of UA buffer and centrifugation twice, 200 μL of 0.5 M tetraethyl ammonium bromide (TEAB) buffer (pH 8.5) were added, followed by 20 min of centrifugation at 14,000× *g*. This procedure was performed twice. The samples were subsequently digested for 16 h at 37 °C, and peptides were extracted by centrifugation at 16,000× *g*. In order to improve peptide yield, the filter was washed twice with 500 μL of 0.5 M TEAB buffer (pH 8.5). Using a vacuum concentrator, the peptide solution was dried.

The CSF peptides were tagged using TMT 10-plexTM label reagent according to the manufacturer’s instructions (Thermo Fisher Scientific, Rockford, IL, USA). Briefly, the TMT labeling reagents were withdrawn from the freezer and brought up to room temperature prior to use (taking approximately 30 min). Then, 41 mL of acetonitrile (ACN) was added to each channel, vortexed to dissolve, centrifuged, and put aside. The reagent was applied to 100 mg of sample solution (i.e., mix 1:1). One hour was given for the solution to stand at room temperature. The reaction was terminated by incubating the solution at room temperature for 15 min after adding 8 mL of hydroxylamine. Each group’s samples from 10 channels were mixed individually and vortexed to ensure thorough mixing. Before freezing at −80 °C, the sample was cleansed of salt and other contaminants.

Using the UPLC 3000 system (Dionex, Sunnyvale, CA, USA) coupled with an XBridgeTM BEH300 C18 column, the peptides were fractionated (Waters, Milford, MA, USA). A was H2O adjusted to a pH of 10 with ammonium hydroxide, and B was acetonitrile adjusted to a pH of 10 with ammonium hydroxide. The following gradient was used to separate peptides: 8 to 18% phase B for 30 min, and 18 to 32% phase B for 22 min. A total of 48 fractions were collected, dried with a speedvac, combined to create 12 fractions, and resuspended with 0.1% formic acid.

The Easy nLC-1000 system, paired with a Q Exactive mass spectrometer, was used to isolate all tagged tryptic peptides (ThermoFisher Scientific) [21]. On a 15 cm column (i.d. 75 um) packed in-house with reverse-phase (RP) materials ReproSil-Pur C18-AQ and 3.0 um resin (Dr. Maisch GmbH, Ammerbuch-Entringen, Germany), peptides were separated. Xcalibur collected data in the data-dependent “top15” mode as follows: 15 most abundant precursor ions in each full scan (MS1 scan: 300–1500 *m*/*z* with resolution 120,000@ *m*/*z* 200, AGC target: 3E6, maximum IT: 50 ms) were selected using an isolation window of 1.0 Da. Additionally, the resolution for MS/MS spectra was set to 60,000 @ *m*/*z* 200, the target value was 1E5 (AGC control enabled, maximum IT: 50 ms), the fragmentation mode was one of higher-energy collision dissociation (HCD), there was a normalized energy of 30%, and dynamic exclusion at 20 s [21]. 

All MS/MS spectra were analyzed using MaxQuant (version 1.6.0.16) to identify peptides and proteins using the UniProt human FASTA protein database (dated 202104, containing 92607 protein entries) [21]. The TMT tags used to label specific amino acid residues, including lysine and peptide N termini (229.162932 Da), along with carbamidomethylation for cysteine residues (57.02146 Da), were treated as static modifications. Methionine residue oxidation was treated as a variable modification. Two missing cleavage sites were allowed. The limits for peptides and fragment ions were established at 10 ppm and 20 ppm, respectively, and strict false discovery rates for peptides and proteins were maintained (not exceeding 0.01). Using reporter ion quantitation, the abundance of each peptide was measured by extracting TMT signals using MaxQuant software. At the same time, precursor ion fraction (75%) limits were applied to minimize co-isolation interference. The relative quantities of each protein were then determined using aggregate peptide data, with normalization conducted under the premise of equal protein loading across all samples.

### 2.3. Enzyme-Linked Immunosorbent Assay (ELISA)

Using ELISA kits, a validation experiment was conducted (ImmunoClone, Huntington Station, NY, USA). The concentrations of insulin-like growth factor-binding protein 7 (IGFBP7) and lysosomal associated membrane protein type-2 (LAMP2) were determined using 120 CSF and plasma samples from 20 MS patients, 20 NMOSD patients, 20 non-inflammatory neurological controls (NINC), and 20 healthy controls (HC). The experiment was conducted in accordance with the manufacturer’s guidelines.

### 2.4. Statistical Analysis

Student’s *t* test and the Mann–Whitney U test were used to compare continuous variables. The test used depended on whether the data were regularly distributed. Using the Spearman rank correlation coefficient, the strength of the link between proteins in serum and CSF was assessed. The diagnostic efficiency was evaluated using the receiver operating characteristic (ROC) area under the curve (AUC). SPSS software (version 19.0; SPSS Inc., Chicago, IL, USA) was used to analyze the data, and GraphPad Prism software (version 5.0; GraphPad Software Inc., San Diego, CA, USA) was used to make the figures. A *p* value of less than 0.05 was deemed to be statistically significant for all two-tailed tests.

## 3. Results

### 3.1. Comparing the Clinical Traits of Patients and Controls

Table 1 displays the clinical features of 80 participants included in the validation cohort. Age and gender did not differ substantially among MS, NMOSD, NINC, and the HC group (*p* > 0.05). In addition, MRI T2 lesion counts were retrieved from the medical records. Disability was scored by a qualified neurologist using the expanded disability status scale (EDSS).

### 3.2. Proteomic Analysis of CSF from MS and NMOSD Patients

**Figure 1:**
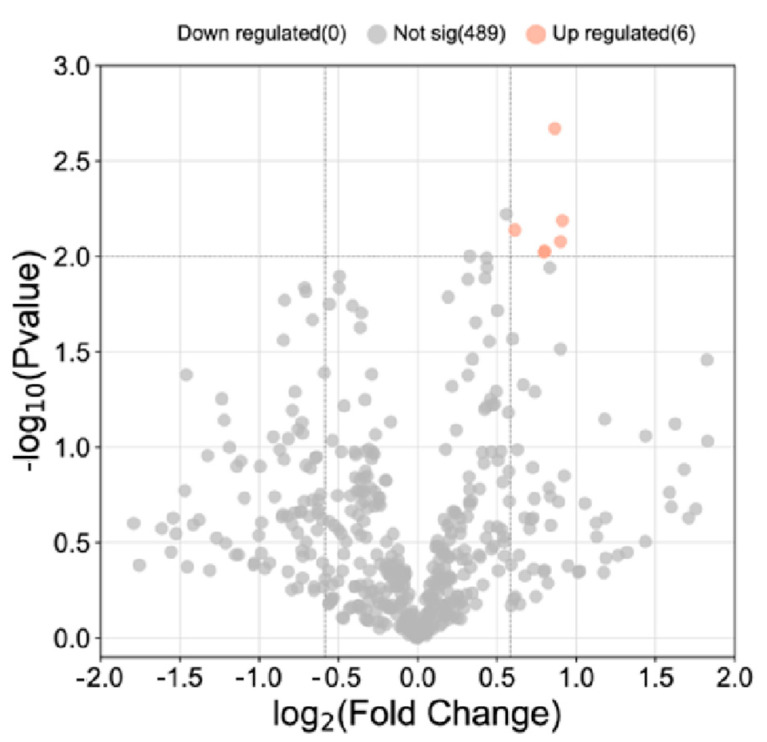
Volcano plot of CFS from 10 MS patients vs. 10 NMOSD patients.

In the discovery cohort, 495 proteins were identified, excluding complement and immunoglobulin. Significantly differentially expressed proteins (DEPs) were selected based on fold change ≥ 1.5 or ≤0.7 and *p* < 0.01 (Figure 1). Six proteins, including transferrin (TF), prosaposin (PSAP), insulin-like growth factor II (IGF2), insulin-like growth factor-binding protein 7 (IGFBP7), lysosome-associated membrane glycoprotein 2 (LAMP2), and EGF-containing fibulin-like extracellular matrix protein 1 (EFEMP1), were found to be differentially expressed between the MS and NMOSD groups. All of these proteins were elevated in MS patients’ CSF. The putatively harmful mechanisms of TF [22,23], PSAP [24,25], and IGF2 [26,27] were documented in several neurodegenerative illnesses. Few reports on EFEMP1 focused on solid tumors [28] and amyloidosis [29,30]. Prior investigations linked IGFBP7 and LAMP2 to demyelinating disease [31] or MS [32], while the underlying mechanism remained unknown. Hence, we selected IGFBP7 and LAMP2 as plausible candidate proteins for further experimental confirmation.

### 3.3. ELISA Validation of Candidate Proteins

**Figure 2:**
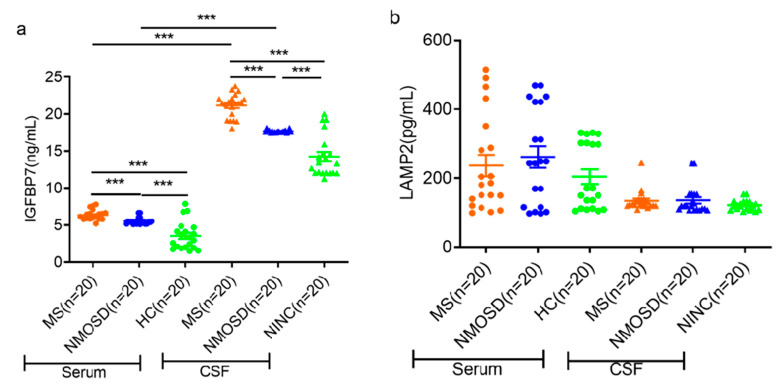
Dot plots depicting the amounts of IGFBP7 and LAMP2 in the serum and CSF of all subjects. (**a**) Measurements of IGFBP7 in the serum and CSF of every individual; (**b**) measurements of LAMP2 in the serum and CSF of every individual. For the sake of brevity, only major variations are displayed. The statistical significance was defined as *** False Discovery Rates < 0.001.

The chosen proteins were confirmed using a commercial ELISA kit and CSF and serum samples from a separate cohort. In comparison to the NMOSD group, the MS group exhibited significantly higher levels of IGFBP7 in both serum and CSF (Figure 2a). This outcome is compatible with our proteomics research findings. In contrast to our proteomic findings, however, there was not a significant change in the amount of LAMP2 protein expression (Figure 2b). As a comparison to the NINC groups, the MS and NMOSD groups had considerably greater concentrations of serum and CSF IGFBP7 (Figure 2a). However, in neither the MS nor the NMOSD groups was there a discernible change in the concentration of LAMP2 in the serum or the CSF (Figure 2b). In addition, patients with MS and NMOSD had lower levels of IGFBP7 in their serum than in their CSF. There was not a discernible difference in LAMP2 levels between the serum and the CSF (Figure 2).

### 3.4. Correlation between Serum and CSF Measurements of Each Protein

**Figure 3:**
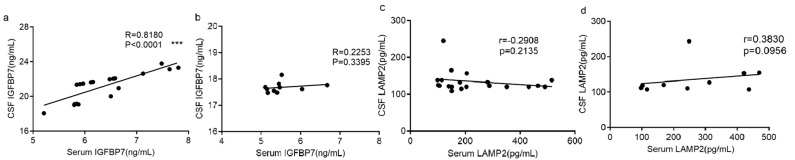
Correlation between these two proteins in MS and NMOSD patients’ serum and CSF. (**a**) Plots illustrating the correlation between CSF IGFBP7 and serum IGFBP7 in 20 MS patients; (**b**) plots illustrating the correlation between CSF IGFBP7 and serum IGFBP7 in 20 NMOSD patients; (**c**) plots illustrating the correlation between CSF LAMP2 and serum LAMP2 in 20 MS patients; (**d**) plots illustrating the correlation between CSF LAMP2 and serum LAMP2 in 20 NMOSD patients. Presented are Pearson’s correlation coefficients (r) and *p* values. The statistical significance was defined as *** p < 0.001.

Figure 3 showed a substantial positive correlation between serum and CSF IGFBP7 in the MS group (r = 0.8, *p* < 0.0001), while there was no such correlation in the NMOSD group. For LAMP2, a correlation between serum and CSF was not found in either the MS group or the NMOSD group (MS: r = −0.3, *p* = 0.214; NMOSD: r = 0.4, *p* = 0.096, Figure 3c,d).

### 3.5. Evaluating the Efficacy of IGFBP7 and LAMP2 in the Diagnosis of MS and NMOSD

**Figure 4:**
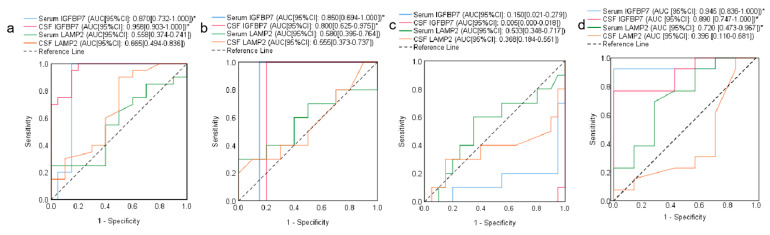
The diagnostic and differentiating value of IGFBP7 and LAMP2. (**a**) ROC curve for MS diagnosis; (**b**) ROC curve for NMOSD diagnose; (**c**) ROC curve for differentiation between NMOSD and MS; (**d**) ROC curve for differentiation between SPMS and RRMS. The statistical significance was defined as * *p < 0.05*.

We used the ROC curve analysis and the Youden index (sensitivity plus specificity minus one) to calculate the optimal diagnostic accuracy of IGFBP7 and LAMP2. For MS diagnosis, serum and CSF IGFBP7 showed excellent AUC above 0.8. The serum IGFBP7 had a diagnostic cutoff of 5.0 ng/mL with 100% sensitivity and 85% specificity, whereas CSF IGFBP7 had a diagnostic cutoff of 17.0 ng/mL with 100% sensitivity and 80% specificity. Both serum LAMP2 and CSF LAMP2 had AUCs below 0.7 and exceedingly low Youden indices. (Figure 4a and Table 2).

For NMOSD diagnosis, serum and CSF IGFBP7 also showed excellent AUC above 0.8. Serum IGFBP7 had a cutoff of 5.0 ng/mL with 100% sensitivity and 85% specificity, whereas CSF IGFBP7 had a cutoff of 16.7 ng/mL with 100% sensitivity and 80% specificity. Conversely, both serum and CSF LAMP2 exhibited extremely low AUC (less than 0.6) and sensitivity (less than 50%, Figure 4b and Table 2).

### 3.6. Effectiveness of IGFBP7 and LAMP2 in Differentiating NMOSD from MS

In addition, the diagnostic performance of these two proteins was evaluated in terms of their ability to differentiate NMOSD from MS. The AUC values for IGFBP7 and LAMP2 were both lower than 0.6. Serum and CSF IGFBP7 showed high sensitivities of 100% but extremely low specificities of 0%. Serum LAMP2 and CSF LAMP2 displayed a poor Youden index of approximately 20% (Table 3 and Figure 4c).

### 3.7. Assessment of the Predictive Capability of IGFBP7 and LAMP2 for SPMS

We also looked into whether or not these two proteins have use as diagnostic markers for SPMS. As depicted in Table 3 and Figure 4d, both serum and CSF IGFBP7 revealed outstanding SPMS prognostic values. CSF IGFBP7 had a cutoff of 21.5 ng/mL with a sensitivity of 76.9% and a specificity of 100%, whereas serum IGFBP7 had a cutoff of 6.0 ng/mL with a sensitivity of 92.3% and a specificity of 100%. The concern is that both serum and CSF IGFBP7 can be used to predict the course of MS, with serum IGFBP7 separating SPMS from RRMS with greater precision than CSF IGFBP7. In addition, under an optimal diagnostic performance, serum LAMP2 showed mild predictive power with a cutoff of 165.7 pg/mL, sensitivity of 69.2%, and specificity of 71.4%, while CSF LAMP2 revealed a low specificity of 14.3%.

## 4. Discussion

The discovery of biomarkers and their clinical implementation has proven to be particularly challenging in the cases of MS and NMOSD. This is likely due to the intricate pathology of these diseases, which includes distinct and frequently coexisting processes, such as inflammation, demyelination, and neurodegeneration [33]. MS often progresses from an early phase of RRMS to a subsequent phase of SPMS [34]. Since the transition from RRMS to SPMS is phenotypically delayed, the decision of clinicians to postpone the diagnosis of SPMS results in an evident, continuous increase in disability. The mean time for RRMS to transition to SPMS diagnostic ambiguity, according to one study, was 3.3 years [35]. Therefore, it is crucial to identify molecular biomarkers for RRMS diagnosis and to monitor the progression of the illness to confirm SPMS.

In this study, we firstly analyzed CSF from individuals with MS and NMOSD using proteomics, and identified two novel possible biomarkers (IGFBP7 and LAMP2) that might be utilized to diagnose NMOSD and MS and to differentiate NMOSD from MS. Subsequently, we examined the diagnostic performance of IGFBP7 and LAMP2 in CSF and serum using the ELISA method on a larger sample size. We demonstrated the excellent sensitivity and specificity of serum IGFBP7 and CSF IGFBP7 for the diagnosis of MS and NMOSD, as well as the outstanding quality of IGFBP7 and serum LAMP2 in forecasting the developing phenotypes of MS. To our knowledge, this is the first study to assess the diagnostic efficiency of these two proteins in both MS and NMOSD patients. 

Consistent with the findings of our proteomics analysis, in the validation cohort we found that serum and CSF IGFBP7 levels in the MS group and NMOSD group were significantly higher than those in the controls, with the MS group exhibiting a greater increase than the NMOSD group. The AUC for CSF IGFBP7 in the MS group was 0.958, which was significantly higher than that seen for serum IGFBP7 (0.870), serum LAMP2 (0.558), and CSF LAMP2 (0.665). In addition, there was a highly significant positive correlation between serum IGFBP7 and CSF IGFBP7 in the MS group (r = 0.8, *p* < 0.0001). Hence, CSF IGFBP7 was necessary for MS diagnosis, but not for both serum IGFBP7 and CSF IGFBP7. 

Excellent AUC values were also found for serum IGFBP7 (0.850) and CSF IGFBP7 (0.800) in the NMOSD group; however, the AUC values were only 0.580 for serum LAMP2 and 0.555 for CSF LAMP2. In contrast to the MS group, no significant association was detected between serum IGFBP7 and CSF IGFBP7 in the NMOSD group. Accordingly, the role of IGFBP7 in the etiology of MS and NMOSD is probably distinct and will require further study if it is to be elucidated. In general, our data demonstrated that IGFBP7 levels in serum and CSF are highly diagnostic for NMOSD.

Although the MS group had significantly greater levels of serum and CSF IGFBP7 than the NMOSD group in both the study cohort and validation cohort, IGFBP7 had an exceedingly low AUC and poor specificity (0%) when differentiating NMOSD from MS. IGFBP7 was deemed to be insufficient for discriminating between these two illnesses.

IGFBP7 is a newly identified member of the superfamily of insulin-like growth factor-binding proteins (IGFBPs), which are expressed in astrocytes, oligodendrocytes, and neurons [31]. IGFBP7 regulates the biological activity of IGFs, which is demonstrated in the development of the central nervous system and the differentiation of oligodendrocyte precursor cells [36,37]. Recent investigations have revealed that IGFBP7 negatively regulates oligodendrocyte differentiation via the Wnt/β-catenin signaling pathway, which is essential for myelin regeneration [38]. In the current investigation, two demyelinating diseases were found to have significantly elevated levels of serum and CSF IGFBP7. Earlier investigations also demonstrated a raised level in pathological CNS disorders, such as glioblastoma and stroke, as well as experimental autoimmune encephalomyelitis (EAE) and MS [21,31,39,40]. In addition, both sera, IGFBP7 and CSF IGFBP7, exhibited great sensitivity and specificity for the diagnosis of these two disorders. Therefore, IGFBP7 may have the potential to serve as a biomarker for diagnosing MS and NMOSD. 

We further evaluated the ability of these two proteins to distinguish SPMS from RRMS. Serum IGFBP7 had a greater AUC (0.945) than CSF IGFBP7 (0.890), serum LAMP2 (0.720), and CSF LAMP2 (0.396), which implies the potential relevance of IGFBP7 in MS categorization; conversely, serum IGFBP7 was more appropriate for use MS phenotypes than CSF IGFBP7 in clinical practice. In general, this result highlights the potential role of IGFBP7 in MS stratification. The association between IGFBP7 expression levels and the degree of inflammatory demyelination [31] possibly explain this outcome. The astonishingly greater level of IGFBP7 in SPMS patients compared to that in RRMS patients may be a result of the disease’s progression bringing an enhanced inflammatory response burden. 

In our investigation, neither serum nor CSF LAMP2 levels were considerably different across groups. The poor performance of LAMP2 in diagnosing MS (serum LAMP2: 0.558; CSF LAMP2: 0.665), diagnosing NMOSD (serum LAMP2: 0.580; CSF LAMP2: 0.555), and distinguishing NMOSD from MS (serum LAMP2: 0.533; CSF LAMP2: 0.368) was evidenced by its low AUC values. It is intriguing that serum LAMP2 had a moderate AUC (0.720) for differentiating SPMS from RRMS.

Lysosomal-associated membrane protein type-2 (LAMP2), a major component of lysosomal membrane proteins, plays a crucial role in autophagy [41]. Numerous studies have shown that autophagy directly influences the progression of MS and EAE [42,43,44]. Previous research has indicated that lysosomal membrane injury leads to the suppression of autophagy and neurodegeneration following brain trauma, as well as to increases in LAMP2 levels in the injured brain section [45]. SPMS patients in our study had greater serum LAMP2 levels than RRMS patients, which could be attributed to the more severe brain damage seen in SPMS. It was hypothesized that LAMP2 could be used as a predictor of MS progression. Nevertheless, LAMP2’s forecast ability was inferior to that of IGFBP7, and further research is required to clarify the function of LAMP2 in the progression of MS.

### Limitations

Our study had some limitations, including that our study excluded patients with cancer and/or cardiac diseases, despite the fact that IGFBP7 expression was observed to be similarly e altered in these patients [46,47]. This factor should be considered in clinical applications. Additionally, additional research is required to determine if these two putative proteins are associated with the other two MS subtypes (primary progressive multiple sclerosis and clinical isolated syndrome). This study provides a preliminary examination of proteins with differential levels of expression in MS and NMOSD. For future elucidation of the underlying mechanisms of MS and NMOSD, it is necessary to collect additional samples to confirm these conclusions and gain a better understanding of the underlying processes of MS and NMOSD.

## 5. Conclusions

As far as we know, this is the first study to evaluate the clinical utility of IGFBP7 and LAMP2 for diagnosing NMOSD and MS. These findings indicated the superior performance of IGFBP7 in efficiently diagnosing MS and NMOSD. Nevertheless, for the diagnosis of MS, only CSF IGFBP7 was necessary. Serum IGFBP7, CSF IGFBP7, and serum LAMP2 had superior performance in forecasting the developing phenotypes of MS, however serum IGFBP7 is preferable. These results provided a rationale for conducting additional research on the roles of IGFBP7 and LAMP2 in the pathogenesis of MS and NMOSD.

## Figures and Tables

**Table 1:** Demographic and clinical information about the research participants.

Variable	MS		NMOSD	NINC	HC
	RRMS (*n* = 7)	SPMS (*n* = 13)			
N (CSF; Serum)	7;7	13;13	20;20	20;0	0;20
Age (year), mean ± SD	38.7 ± 12.5	33.1 ± 11.4	36.6 ± 10.0	37.2 ± 10.4	38.5 ± 8.0
Male (%)	3(42.9%)	5(38.5%)	8(40.0%)	7(35.0%)	7(35.0%)
Disease duration (year), mean ± SD	3.1 ± 1.3	13.8 ± 5.9	10.1 ± 7.1	-	-
EDSS, mean ± SD	2.5 ± 1.2	4.1 ± 1.7	-	-	-
MRI lesion			-	-	-
0–8 lesions, *n*, *n*%	9, 45.0%	8, 40.0%			
≥9 lesions, *n*, *n*%	11, 55.0%	12, 60.0%			

N: number; RRMS: relapsing remitting multiple sclerosis; SPMS: secondary progressive multiple sclerosis; NINC: non-inflammatory neurological controls; HC: healthy controls; EDSS: expanded disability status scale.

**Table 2:** Diagnostic Value of IGFBP7 and LAMP2 for MS and NMOSD Diagnosis.

Biomarkers	MS vs. Control			NMOSD vs. Control		
	Cut-off Point	Sensitivity	Specificity	Cut-Off Point	Sensitivity	Specificity
Serum IGFBP7	5.0	100%	85%	5.0	100%	85%
CSF IGFBP7	17.0	100%	80%	16.7	100%	80%
Serum LAMP2	341.5	25%	100%	376.5	30%	100%
CSF LAMP2	119.4	90%	50%	154.4	20%	100%

**Table 3:** The diagnostic value of IGFBP7 and LAMP2 in the differential diagnosis of MS and NMOSD, as well as the distinction between SPMS and RRMS.

Biomarkers	MS vs. NMOSD			RRMS vs. SPMS		
	Cut-Off Point	Sensitivity	Specificity	Cut-Off Point	Sensitivity	Specificity
Serum IGFBP7	4.1	100%	0%	6.0	92.3%	100%
CSF IGFBP7	16.5	100%	0%	21.5	76.9%	100%
Serum LAMP2	224.5	60%	65%	165.7	69.2%	71.4%
CSF LAMP2	146.0	30%	85%	111.7	100%	14.3%

## Data Availability

The data presented in this study are available on reasonable request from the corresponding author. The datasets presented in this study can be found in online repositories. The names of the repositories and accession numbers can be found below: http://www.proteomexchange.org/, IPX0002604000, PXD023027, accessed on 17 August 2021.
